# Repair Performance of Additively and Subtractively Manufactured Permanent Crown Materials: A Combined Mechanical and Optical Evaluation

**DOI:** 10.3390/ma19112406

**Published:** 2026-06-05

**Authors:** İrem Karagözoğlu, Özge Parlar Öz, Nermin Demirkol, Tan Fırat Eyüboğlu, Mutlu Özcan

**Affiliations:** 1 Department of Prosthodontics, Faculty of Dentistry, Gaziantep University, Gaziantep 27310, Türkiyedt_nerminhamdemirci@hotmail.com (N.D.); 2 Department of Endodontics, Faculty of Dentistry, Istanbul Medipol University, Istanbul 34083, Türkiye; tfeyuboglu@yahoo.com; 3 Clinic of Masticatory Disorders and Dental Biomaterials, Center for Dental Medicine, University of Zurich, 8032 Zurich, Switzerland; mutlu.ozcan@zzm.uzh.ch

**Keywords:** 3D printing, CAD/CAM materials, dental materials, repair bond strength, tribochemical silica coating, color difference, resin-matrix restorations

## Abstract

**Highlights:**

- CAD/CAM materials showed higher repair bond strength than 3D-printed resins.
- Tribochemical silica coating yielded the highest bond strength across all groups.
- Additively manufactured materials exhibited greater color change after aging.
- Aging increased color mismatch, but values remained clinically acceptable.
- Repair performance depends on both material type and surface treatment.

**Abstract:**

This study aimed to investigate the repair performance of these materials subjected to different surface treatment protocols using combined mechanical and optical evaluations. Four resin-matrix materials were evaluated: two CAD/CAM materials (HIPC Plus and GC Cerasmart) and two additively manufactured resins (VarseoSmile TriniQ and CrownTec). Standardized disc specimens were fabricated and thermocycled prior to repair. Three surface treatments were applied: airborne particle abrasion (APA), tribochemical silica coating (TSC) with silane, and laser treatment (LT). Micro-shear bond strength (µSBS) was measured using composite cylinders bonded to treated surfaces. For optical evaluation, standardized cavities were restored with composite resin, and color measurements were obtained at baseline (T0) and after aging (T1) using a spectrophotometer. Color mismatch and color stability were calculated. Data were analyzed using two-way ANOVA and Tukey tests (α = 0.05). Material type and surface treatment significantly affected µSBS (*p* < 0.001). CAD/CAM materials had higher bond strength than additively manufactured ones. The TSC group showed the highest µSBS. Aging increased color mismatch, with post-aging ΔE00 values surpassing clinical thresholds. Additively manufactured materials experienced greater color changes. Repair performance depends on manufacturing method and surface treatment. The higher bond strength in the TSC group likely results from silica coating and silanization. CAD/CAM materials showed better optical stability.

## 1. Introduction

Advances in digital technologies have enabled the widespread use of subtractive (CAD/CAM) and additive (3D printing) methods in restorative dentistry. CAD/CAM resin-matrix ceramics and hybrid blocks are polymerized under industrial conditions, resulting in homogeneous structures and predictable properties, whereas 3D-printed permanent crown resins are produced via layered photopolymerization and post-curing, leading to variations in surface chemistry and bonding [1]. Both methods yield biocompatible, clinically acceptable restorations; however, long-term complications such as chipping, marginal deterioration, wear, or discoloration may occur due to patient-related factors or functional loading [2,3].

3D-printed resins are particularly susceptible to localized damage because of their microstructural characteristics [4,5]. Layered fabrication and variations in the degree of conversion can introduce internal defects and weak interlayer bonding, facilitating fracture under load [6,7]. Their lower hardness and elastic modulus may further increase the risk of deformation and surface damage [6]. Environmental factors such as water exposure, thermocycling, and fatigue promote degradation and crack propagation [8]. Chipping is not limited to additive materials; CAD/CAM ceramics and nanoceramics are also affected, with reported incidences of 4–10% in bilayered restorations, particularly under tensile stresses or in the presence of structural flaws [9]. Additionally, repeated loading and aging can induce microcracks and phase changes, increasing susceptibility to fracture over time [10,11,12,13].

In permanent restorations, minor cracks or chipping may not require immediate replacement and can be monitored or repaired. Intraoral repair is a clinically advantageous alternative to full replacement for defects such as chipping, marginal breakdown, wear, or localized damage, reducing treatment time and cost while supporting minimally invasive approaches [14,15,16]. Its success primarily depends on adhesion between the existing restoration and the repair composite, which is influenced by surface treatment, adhesive systems, and repair materials [16,17]. Bonding relies on micromechanical interlocking and chemical adhesion to the organic matrix and fillers [14,18]. Mechanical pretreatments, including bur roughening, airborne-particle abrasion (Al_2_O_3_), silica-coated particles, laser irradiation, and acid etching, enhance surface roughness and interlocking [16,19]. Among these, airborne-particle abrasion is widely preferred due to its consistent roughening effect and its ability to improve retention and bond strength in resin-based substrates [16,20], as well as surface roughness and shear bond strength in CAD/CAM materials [21]. Chemical pretreatments such as silanes, primers, and adhesives further enhance bonding effectiveness [22].

The success of an intraoral repair relies on both restoring mechanical strength and achieving acceptable optical results [23], as variations in color match and optical stability can occur over time. Limited evidence exists on optical outcomes post-repair, especially for materials made by different techniques. However, assessing changes in color and translucency after staining or aging offers clinically relevant insights into the long-term esthetic acceptability of repairs [23,24].

Despite the growing number of studies on the repair bond strength of indirect restorative materials, most focus on mechanical performance, with limited attention to optical outcomes [19,20,21,22]. Few have directly compared repair results between additively and subtractively manufactured crown materials under standardized protocols [14,16,23]. With the rise of hybrid CAD/CAM and 3D-printed resins, there is a need for studies assessing both mechanical durability and esthetic integration of repairs within a single experimental design. Therefore, the aim of the present in vitro study was to evaluate the repair performance of additively and subtractively manufactured permanent crown materials following different surface treatments, using a combined mechanical (micro-shear bond strength) and optical (color difference) assessment. The null hypotheses tested were that (1) surface treatment protocols would not affect repair bond strength, (2) the type of restorative material would not influence repair bond strength, and (3) material type would not affect color difference (ΔE00).

## 2. Materials and Methods

This study was designed to evaluate the repair performance of additively and subtractively manufactured permanent crown materials following different surface treatments. Sample size calculation was performed using G*Power software (version 3.1, Heinrich-Heine-Universität Düsseldorf, Düsseldorf, Germany). The statistical power was set at 80%, with a significance level of α = 0.05 and a two-tailed hypothesis. Based on previous studies evaluating repair bond strength of resin-based restorative materials, a large effect size (f = 0.55) was used for the sample size calculation [14,16]. According to the power analysis, a minimum of 10 specimens per subgroup was considered sufficient for statistical analysis. The sample size calculation was based on the primary mechanical outcome (micro-shear bond strength); therefore, the optical outcomes were considered secondary exploratory outcomes and interpreted accordingly. Two independent specimen sets were prepared for mechanical and optical evaluations because micro-shear bond strength testing is a destructive procedure that alters the specimen surface and may influence optical measurements. The first set (Set A) of experiments was designed to assess the micro-shear bond strength (μSBS) and failure mode analysis. The second set (Set B) of experiments focused on the color difference (ΔE00). Each experimental group comprised thirty specimens (n = 30). The following four permanent crown materials were investigated: Group 1 (HIPC); HIPC Plus (breCAM.HIPC, bredent GmbH & Co., Ltd., Senden, Germany), Group 2 (GC); GC Cerasmart (GC Corp., Tokyo, Japan), Group 3 (VST); VarseoSmile TriniQ (BEGO, Bremen, Germany), and Group 4 (CT); CrownTec (Saremco Dental, Rebstein, Switzerland). The materials used in this study, their composition, and manufacturers are presented in Table 1.

### 2.1. Specimen Preparation

Standardized disc-shaped specimens (Ø10 mm × 2 mm) were digitally designed in standard tessellation language (STL) format using 3D modeling software (Exocad Elefsina v3.2, Exocad GmbH, Darmstadt, Germany) to ensure uniform specimen geometry across all materials. The STL file was used to fabricate specimens from CAD/CAM blocks (n = 120) and 3D-printed materials (n = 120) according to manufacturers’ instructions. All additively manufactured specimens were fabricated using a digital light processing (DLP) 3D printer (DentaFab Sega 3D Printer, DentaFab, Istanbul, Turkey). The STL files were imported into the DentaFab printer software (DentaFab, Istanbul, Turkey; https://www.dentafab.com/, accessed on 20 February 2026) and printed in a horizontal orientation (0°) with a layer thickness of 50 µm using the manufacturer-recommended resin profile and printing parameters. Support structures were not used because of the flat disc-shaped specimen geometry. The printing process was based on layer-by-layer photopolymerization of methacrylate-based permanent crown resins, with each layer selectively polymerized using projected light according to the STL design file. After printing, the specimens were washed in a washing unit (Medifive Twin Cure, Medifive Co., Ltd., Incheon, Republic of Korea) containing 95% ethanol for 5 min, in accordance with the manufacturer’s instructions, to remove uncured resin residues. The specimens were then gently air-dried using oil-free compressed air. Subsequently, post-polymerization was performed in a UV light curing unit (Medifive Twin Cure, Medifive Co., Ltd., Republic of Korea) with a curing wavelength range of 365–405 nm for 30 min to enhance the degree of conversion and improve the mechanical stability of the printed specimens.

In the subtractive specimen group, the STL files were transferred to a 5-axis milling unit (inLab MC X5; Dentsply Sirona, Bensheim, Germany), and the specimens were milled from pre-polymerized CAD/CAM blocks in accordance with the manufacturer’s instructions. After fabrication, all specimens were embedded in auto polymerizing acrylic resin using a custom-made stainless-steel mold to facilitate handling and standardization during subsequent procedures. Each specimen was positioned in the center of the mold with its test surface oriented parallel to and flush with the acrylic resin surface, ensuring a standardized bonding area.

**Figure 1:**
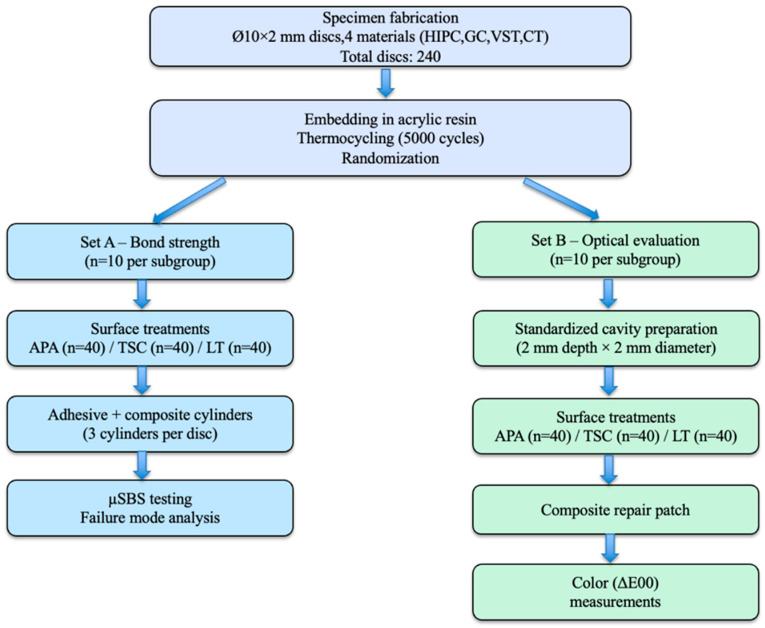
Experimental design and specimen allocation flowchart.

To simulate intraoral aging prior to repair, the embedded specimens were thermocycled in distilled water between 5 °C and 55 °C, with a 30-s dwell time and a 10-s transfer time, for a total of 5000 cycles (SD Mechatronik Thermocycler; SD Mechatronik GmbH, Feldkirchen-Westerham, Germany). This aging protocol was intended to simulate thermal stresses and water exposure in the oral environment, which may affect the bonding performance of repaired restorations. After aging, the bonding surfaces were lightly wet-ground with silicon carbide abrasive papers to standardize the surface and remove superficial contaminants. The specimens were then steam-cleaned and dried with oil-free compressed air before further surface treatment. A schematic overview of the experimental design and specimen allocation is presented in Figure 1.

### 2.2. Surface Treatment Protocols

Specimens allocated to Set A were randomly assigned using a computer-generated randomization sequence to one of three surface treatment protocols prior to the repair procedure. In the airborne particle abrasion (APA) subgroup, surfaces were treated with 50-µm Al_2_O_3_ particles using an intraoral sandblasting device (CoJet Prep; 3M ESPE, Seefeld, Germany) at 2 bar pressure for 10 s from a distance of approximately 10 mm. In the tribochemical silica coating (TSC) subgroup, surfaces were sandblasted with 45-µm silica-coated aluminum oxide particles (CoJet Sand; 3M ESPE, Seefeld, Germany) using the CoJet System at a distance of 10 mm, applied perpendicular to the bonding surface under 2.8 bar pressure for 20 s [25]. After sandblasting procedures, all specimens were thoroughly rinsed with distilled water, ultrasonically cleaned in distilled water for 5 min to remove residual abrasive particles, and dried with oil-free compressed air before bonding procedures. In the laser treatment (LT) subgroup, surface conditioning was performed using an Er:YAG laser (PDT Combine, Fotona, Gruibingen, Germany). The laser parameters were selected based on previous studies reporting effective surface modification of resin-based restorative materials without excessive thermal damage [26]. The laser was operated at an output power of 1.5 W, pulse energy of 150 mJ, and a wavelength of 2940 nm. Laser irradiation was applied to each specimen for 20 s at a working distance of approximately 1.5 cm, with the laser tip positioned perpendicular to the specimen surface at an angle of 90°. Irradiation was performed under air–water cooling, and the laser tip was moved in a scanning motion to ensure uniform surface treatment. After laser irradiation, all specimens were rinsed with water and air-dried prior to bonding procedures.

In Set B, a standardized circular cavity was prepared in the center of each disc prior to surface treatment to simulate a localized clinical defect. The cavity was prepared using a cylindrical diamond bur under water cooling to a standardized depth and diameter (2 mm depth and 2 mm diameter). After cavity preparation, the specimens were rinsed with water and gently air-dried to remove debris prior to surface treatment. Specimens were randomly assigned to the same three surface treatment protocols described for Set A: airborne particle abrasion (APA), tribochemical silica coating (TSC), and laser surface treatment (LT). After surface treatment, all specimens were rinsed with water and air-dried prior to bonding procedures. All treatments were performed by a single operator to ensure standardization.

### 2.3. Repair Procedure

After surface treatment, specimens were subjected to the repair procedure. In the tribochemical silica coating (TSC) group, a silane coupling agent (Clearfil Ceramic Primer Plus, Kuraray Noritake Dental, Tokyo, Japan) was applied to the treated surface using a microbrush, allowed to react for 60 s, and gently air-dried. It should be noted that silanization was applied only in the TSC group as part of the tribochemical silica coating protocol. No silanization was performed in the airborne particle abrasion (APA) or laser treatment (LT) groups. Subsequently, a universal adhesive system (Clearfil Bond Quick 2, Kuraray Noritake Dental, Japan) was applied to all specimens according to the manufacturer’s instructions. The adhesive was actively rubbed onto the surface using a microbrush, followed by gentle air-drying to evaporate solvents and obtain a uniform adhesive layer. The adhesive was then light-cured for 10 s using an LED curing unit (Bluephase; Ivoclar AG, Schaan, Liechtenstein) positioned perpendicular to the surface at a standardized distance in high power mode (light power density: 950 mW/cm^2^). A nanohybrid resin composite (Clearfil Majesty ES-2 Classic, Kuraray, Tokyo, Japan) was used as the repair material for all specimens.

### 2.4. Micro-Shear Bond Strength (µSBS) Testing

For micro-shear bond strength testing, transparent cylindrical molds (Tygon tubing, Saint-Gobain Performance Plastics, Courbevoie, France) with an internal diameter of 1 mm and a height of approximately 2 mm were positioned on the treated surfaces to standardize the dimensions of the composite cylinders (Clearfil Majesty ES-2 Classic, Kuraray, Tokyo, Japan). Three molds were placed on each disc with adequate spacing between cylinders. The composite resin was placed into the molds and light-cured according to the manufacturer’s instructions. After polymerization, the molds were carefully removed, and the specimens were stored in distilled water at 37 °C for 24 h prior to testing. Each composite cylinder bonded to the specimen surface was loaded individually using a universal testing machine (Shimadzu AG-XD 50kN, Shimadzu Corporation, Kyoto, Japan) equipped with a micro-shear test device. A thin orthodontic wire (approximately 0.20–0.25 mm in diameter) was looped around the base of the composite cylinder and positioned as close as possible to the cylinder–substrate interface without contacting the surrounding surface. Load was applied at a crosshead speed of 1 mm/min until failure occurred. The maximum load at failure (N) was recorded, and bond strength values (MPa) were calculated by dividing the failure load by the bonded area (A = πr^2^), where r is the radius of the composite cylinder (r = 0.5 mm for a 1-mm diameter cylinder). Since three cylinders were prepared on each disc, the mean µSBS value of the three cylinders was calculated for each disc. The disc, rather than the individual composite cylinder, was considered the experimental unit for statistical analysis to avoid pseudoreplication. After debonding, failure modes were examined under a stereomicroscope (M420; Leica, Wetzlar, Germany) at ×20–×40 magnification and classified as adhesive (failure at the interface), cohesive (within the substrate or composite), or mixed (combination of adhesive and cohesive features).

### 2.5. Optical Evaluation

In Set B, the prepared cavities were restored using the same composite resin. The same shade (A1) was selected for all materials to standardize optical evaluation. Following cavity preparation, specimens underwent the assigned surface treatment protocols. In the tribochemical silica coating group, a silane coupling agent (Clearfil Ceramic Primer Plus, Kuraray Noritake Dental, Japan) was applied according to the manufacturer’s instructions. Subsequently, a universal adhesive system (Clearfil Bond Quick 2, Kuraray Noritake Dental, Japan) was actively applied to the treated surfaces using a microbrush, gently air-dried to evaporate solvents, and light-cured. The composite resin was then placed incrementally into the cavities to minimize void formation and light-cured according to the manufacturer’s instructions. After polymerization, finishing and polishing were performed using a multi-step polishing system (Sof-Lex discs, 3M ESPE, St. Paul, MN, USA) under water cooling. Medium, fine, and superfine discs were sequentially applied for approximately 15 s per step at 10,000 rpm, using light and intermittent pressure. The discs were applied parallel to the specimen surface in a circular motion to standardize the polishing direction and minimize heat generation. After polishing, specimens were thoroughly rinsed and dried with oil-free air. Each specimen was stored separately in an individual sealed container containing distilled water at 37 °C for 24 h before color measurements. All finishing and polishing procedures were performed by a single operator to ensure standardization.

Color measurements were performed using a spectrophotometer (VITA EasyShade, VITA Zahnfabrik, Bad Säckingen, Germany) under standardized conditions (D65 illuminant and 10° observer angle). The device was calibrated before each measurement session according to the manufacturer’s instructions. For each specimen, color coordinates (L*, a*, b*) were recorded from two standardized areas: the substrate surface adjacent to the repair (reference) and the composite repair area. The measuring tip was positioned perpendicular to the surface, and three consecutive readings were taken from each area; mean values were used for analysis. Measurements were performed after 24 h of storage in distilled water at 37 °C (baseline, T0) and repeated after the aging protocol (T1). After baseline (T0) measurements, all specimens were subjected to an artificial aging protocol consisting of 5000 thermocycles in distilled water between 5 °C and 55 °C, with a dwell time of 30 s and a transfer time of 10 s, using a thermocycling device (SD Mechatronik Thermocycler; SD Mechatronik GmbH, Feldkirchen-Westerham, Germany). No additional staining, brushing simulation, or mechanical fatigue procedures were performed during the aging process. The color mismatch between the repair and substrate surfaces at each time point was calculated using the CIEDE2000 formula (ΔE00_mismatch = ΔE00[Sub vs. Rep]). In addition, the color stability of the repair area and substrate area over time was calculated as ΔE00_repair change = ΔE00[Rep T1 vs. Rep T0] and ΔE00_substrate change = ΔE00[Sub T1 vs. Sub T0]. All measurements were performed against a neutral gray background to minimize the influence of external light and background color and were performed by a single operator to ensure consistency.

### 2.6. Statistical Analysis

Descriptive statistics were presented as mean and standard deviation for numerical variables and as frequency and percentage for categorical variables. The normality of micro-shear bond strength and color data was assessed using the Shapiro–Wilk test. Micro-shear bond strength values were analyzed using two-way ANOVA to evaluate the effects of material type, surface treatment, and their interaction. Color mismatch values at baseline (T0) and after aging (T1) were analyzed separately using two-way ANOVA. In addition, color-change variables, including ΔE00_RepairChange and ΔE00_SubChange, were analyzed using two-way ANOVA to evaluate aging-related changes in the repair and substrate areas. When significant differences were detected, Tukey’s post hoc test was used for pairwise comparisons. Failure mode distributions were evaluated descriptively using frequency and percentage values, and no inferential statistical analysis was performed for failure mode distribution. All statistical analyses were performed using SPSS software (version 22.0; IBM Corp., Chicago, IL, USA), and the significance level was set at *p* < 0.05.

## 3. Results

### 3.1. Micro-Shear Bond Strength

Two-way ANOVA demonstrated that micro-shear bond strength (µSBS) was significantly affected by both the type of restorative material and the surface treatment protocol (*p* < 0.001). A significant main effect was observed for material (F = 198.880, *p* < 0.001, η^2^ = 0.847) and surface treatment (F = 193.876, *p* < 0.001, η^2^ = 0.782). Furthermore, a statistically significant interaction between material and surface treatment was identified (F = 12.739, *p* < 0.001, η^2^ = 0.414), indicating that the effect of surface conditioning depended on the material type (Table 2).

The bond strength values of HIPC (18.13 ± 1.91 MPa) and GC Cerasmart (18.08 ± 1.81 MPa) were significantly higher than those of CrownTec (14.65 ± 2.24 MPa) and VarseoSmile TriniQ (13.99 ± 1.57 MPa) (*p* < 0.001). When the results of the surface treatments were analysed together, the tribochemical silica coating group, in which silanization was additionally applied, produced significantly higher bond strength values (18.38 ± 1.86 MPa) than airborne particle abrasion (APA) (14.96 ± 2.77 MPa) or laser treatment (LT) (15.30 ± 1.86 MPa) (*p* < 0.001). However, no significant difference was observed between APA and LT (*p* > 0.05) (Table 3).

**Figure 2:**
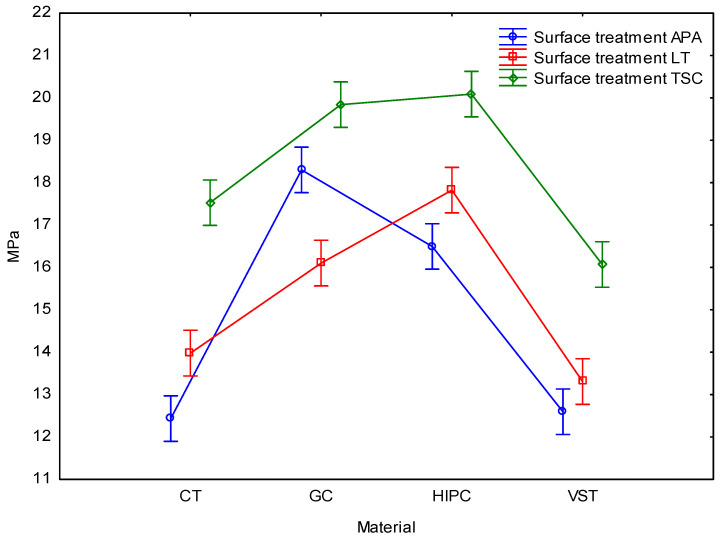
The graphical representation shows mean micro-shear bond strength values for each material according to the surface treatment protocol. CT: CrownTec; VST: VarseoSmile TriniQ; HIPC: HIPC Plus; GC: GC Cerasmart.

Material–surface treatment comparisons revealed that the TSC group with silanization produced the highest µSBS values for all tested materials. For CrownTec and VarseoSmile TriniQ, TSC with silanization resulted in significantly higher bond strength than both APA and LT (*p* < 0.001), while no difference was observed between APA and LT. For HIPC, TSC with silanization showed significantly higher bond strength than APA and LT (*p* < 0.001), and LT also showed significantly higher bond strength than APA (*p* < 0.05). For GC Cerasmart, TSC with silanization yielded significantly higher bond strength than LT (*p* < 0.001), and APA showed significantly higher values than LT (*p* < 0.001) (Table 3, Figure 2).

### 3.2. Failure Mode Analysis

The distribution of failure modes varied according to both material type and surface treatment protocol (Table 4). Overall, mixed failures were the most frequently observed pattern of failure across all materials. VarseoSmile TriniQ showed no adhesive failures, with most failures classified as mixed. In contrast, adhesive failures were more frequently observed in the APA groups, while the TSC groups exhibited a higher proportion of cohesive failures. Notably, the TSC group, which included silanization, was associated with an increased incidence of cohesive failures, whereas airborne particle abrasion was associated with a greater frequency of adhesive failures. These findings are consistent with the bond strength results, indicating that stronger adhesion was associated with a shift from adhesive to cohesive or mixed failure patterns. Failure mode data were evaluated descriptively, and no inferential statistical comparison was performed.

### 3.3. Color Mismatch (ΔE00)

At baseline (T0), the mean color mismatch (ΔE00) between the repair and substrate surfaces was 1.49 ± 0.33, indicating a noticeable but acceptable color difference. Two-way ANOVA revealed no significant main effects of material type or surface treatment on ΔE00 mismatch T0 values (*p* = 0.992 and *p* = 0.806, respectively). However, a statistically significant interaction between material type and surface treatment was observed (*p* = 0.006, partial eta squared = 0.151), although the model’s overall explanatory power was low (adjusted R^2^ = 0.069) (Table 5). Post hoc comparisons did not demonstrate consistent pairwise differences between subgroups. These findings suggest that prior to aging, the initial color compatibility between the repair and substrate surfaces was similar for all materials and surface treatment protocols.

After aging (T1), the mean color mismatch (ΔE00) between the repair and substrate surfaces increased to 2.35 ± 0.29, exceeding the commonly reported clinical acceptability threshold. Two-way ANOVA showed that there was no significant main effect of material type (*p* = 0.354) or surface treatment (*p* = 0.534) on the T1 mismatch values. Additionally, no significant interaction between material and surface treatment was observed (*p* = 0.568) (Table 5). These results suggest that aging caused a similar increase in color mismatch for all materials and surface treatment protocols tested. To clarify the origin of this increase in mismatch, color stability analyses were performed.

### 3.4. Color Stability (ΔE00_RepairChange and ΔE00_SubChange)

**Figure 3:**
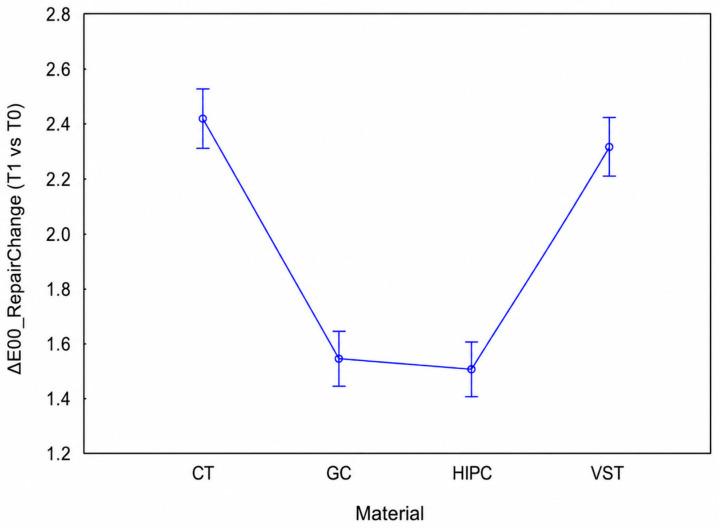
Color change of the repair area after aging (ΔE00_RepairChange) for each material group. Additively manufactured materials (CrownTec and VarseoSmile TriniQ) exhibited significantly higher color change compared with CAD/CAM materials (HIPC Plus and GC Cerasmart) (*p* < 0.001). CT: CrownTec; VST: VarseoSmile TriniQ; HIPC: HIPC Plus; GC: GC Cerasmart.

The color change of the repair surface after aging (ΔE00_RepairChange) was significantly influenced by material type (*p* < 0.001, η^2^ = 0.738), while surface treatment had no significant effect (*p* = 0.439). CrownTec (2.41 ± 0.32) and VarseoSmile TriniQ (2.32 ± 0.28) exhibited significantly higher color change values compared to GC Cerasmart (1.54 ± 0.22) and HIPC (1.50 ± 0.22) (Figure 3).

**Figure 4:**
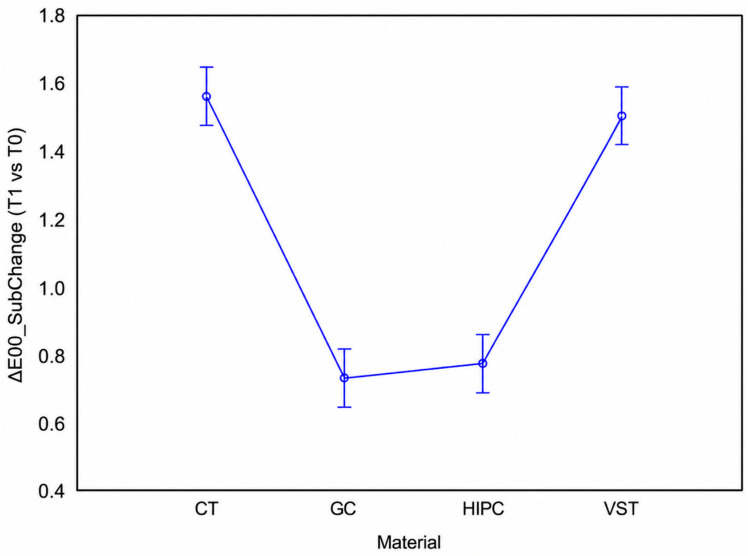
Color change of the substrate after aging (ΔE00_SubChange) for each material group. Additively manufactured materials (CrownTec and VarseoSmile TriniQ) exhibited significantly higher color change compared with CAD/CAM materials (HIPC Plus and GC Cerasmart) (*p* < 0.001). CT: CrownTec; VST: VarseoSmile TriniQ; HIPC: HIPC Plus; GC: GC Cerasmart.

Similarly, substrate color change (ΔE00_SubChange) was significantly affected by material type (*p* < 0.001, η^2^ = 0.741). The 3D-printed materials (CrownTec and VarseoSmile TriniQ) demonstrated significantly higher color change compared with CAD/CAM materials (GC Cerasmart and HIPC) (Figure 4).

These findings suggest that the increase in color mismatch associated with aging was primarily due to the intrinsic color instability of the repair materials, particularly in additively manufactured resins.

## 4. Discussion

The present study evaluated the repair bond strength and color stability of permanent crown materials manufactured using different surface treatment protocols. The null hypothesis that neither material type nor surface treatment would affect repair performance was partially rejected. Although surface treatment significantly influenced micro-shear bond strength, material type was found to play a significant role in determining both bond strength and color stability outcomes. However, surface treatment did not significantly affect color mismatch or color stability after aging. Accordingly, the mechanical and optical findings should be interpreted independently, as they were influenced by different underlying factors.

Ceramic materials are widely used for definitive restorations due to their excellent esthetic properties and high mechanical strength; however, their clinical repair procedures can be challenging [27]. In cases of chipping, marginal defects, or localized surface damage, ceramic restorations often require complex surface conditioning protocols, and intraoral repair may be less predictable compared with resin-based materials [28]. In contrast, resin-matrix restorative materials demonstrate more favorable repairability because of their polymeric matrix, which enables micromechanical retention and chemical bonding with resin composites [16]. This characteristic allows chairside intraoral repair procedures to be performed more easily and conservatively without removing the entire restoration [29]. Moreover, resin-matrix CAD/CAM materials exhibit mechanical behavior that more closely resembles that of natural tooth structures, including a relatively lower modulus of elasticity and improved stress distribution, which may reduce catastrophic failure and facilitate repair procedures [30]. For these reasons, resin-matrix materials have been increasingly investigated in studies focusing on repair performance, bonding behavior, and long-term clinical maintenance of restorative materials [31,32]. Therefore, the present study specifically focused on resin-based restorative materials manufactured through both subtractive and additive technologies. Clinically, this approach supports conservative management strategies by enabling repair instead of full replacement in cases of localized defects.

Resin-matrix restorative materials can be produced using either subtractive milling or additive manufacturing technologies, and the fabrication method may influence their microstructure and overall performance [33]. In subtractive manufacturing, restorations are milled from pre-polymerised industrial blocks produced under highly controlled temperature and pressure conditions, resulting in a highly cross-linked polymer network with homogeneous filler distribution and high monomer conversion [34]. In contrast, additive manufacturing of resin materials involves layer-by-layer photopolymerisation processes, followed by post-curing to enhance polymerisation [35]. However, this incremental fabrication process can introduce interlayer interfaces, variations in polymer network density, and differences in degree of conversion compared with CAD/CAM blocks [36]. These microstructural differences provide a plausible explanation for the inferior bond strength and greater color instability observed in the additively manufactured materials.

Surface treatment plays a crucial role in the repair bond strength of resin-matrix restorative materials. In the present study, the tribochemical silica coating protocol, in which silanization was additionally applied, produced the highest micro-shear bond strength values across all tested materials. Similar findings have been reported in several studies investigating the repair bond strength of CAD/CAM resin-based restorative materials [37,38,39,40,41,42,43]. This outcome can be explained by the combined contribution of micromechanical retention and chemical bonding, which together enhance interfacial stability. The higher bond strength observed in the tribochemical silica coating group should be interpreted as the combined effect of silica coating and silanization, since silane was applied only in this group as part of the clinical tribochemical silica coating protocol. The significant interaction between material type and surface treatment further indicates that bonding effectiveness is material dependent, suggesting that a single surface conditioning protocol may not be equally effective for all restorative materials.

The analysis of failure modes provided additional insight into the bonding performance of the tested surface treatment protocols. Generally, groups with higher bond strength values exhibited a greater proportion of mixed and cohesive failures, while lower-performing groups showed predominantly adhesive failures [44]. In the present study, the predominance of mixed failures in groups with higher bond strength supports the formation of a more stable bonding interface. However, as failure modes were assessed descriptively without inferential statistics, these observations should be interpreted as supportive rather than definitive evidence.

Color differences were assessed using established CIEDE2000 perceptibility and acceptability thresholds of 0.8 and 1.8, respectively, as reported by Paravina et al. [45]. These thresholds were used as reference values for interpreting both repair–substrate color mismatch and aging-related color change outcomes. The results showed that color mismatch increased after aging and exceeded clinically acceptable thresholds, particularly in additively manufactured materials. Given that neither material type nor surface treatment significantly influenced post-aging mismatch values, this increase appears to reflect a general aging-related effect rather than a treatment-specific outcome. The observed color changes are likely related to intrinsic material characteristics, including polymer network structure, degree of conversion, and filler distribution, as well as water absorption and matrix degradation during aging. The low adjusted R^2^ values further indicate that these variables alone do not fully explain the observed optical changes, suggesting the involvement of additional material-related factors. However, because color mismatch values at T0 and T1 were analyzed separately rather than using a repeated-measures model including time as a within-specimen factor, aging-related color changes should be interpreted with caution.

Previous studies comparing additively and subtractively manufactured materials have reported variable outcomes regarding color stability [24,46,47]. In the present study, additively manufactured materials exhibited greater color changes after aging than CAD/CAM materials. This finding supports the view that material composition and manufacturing process play a more decisive role in long-term optical behavior than surface treatment protocols. The higher color change observed in the additively manufactured materials may also be related to differences in post-polymerization behavior and water interaction, although these mechanisms were not directly measured in the present study and should therefore be interpreted cautiously.

The present study provides valuable findings; however, several limitations should be considered. First, this investigation was conducted under in vitro conditions and does not fully replicate the clinical environment. No post-repair thermomechanical aging was performed; therefore, the bond strength results represent short-term repair performance. Additionally, only a limited number of materials and surface treatment protocols were evaluated. Because silanization was applied only in the tribochemical silica coating group, the individual contributions of silica coating and silane application could not be distinguished. The optical outcomes should also be interpreted with caution, as the sample size calculation was based on the mechanical outcome. No direct surface characterization analyses, such as surface roughness measurement, SEM, profilometry, contact angle, or surface chemistry evaluation, were performed. Therefore, the proposed mechanisms related to micromechanical retention, silica deposition, and laser-induced surface modification should be interpreted as possible explanations rather than direct evidence. Future studies should focus on identifying additional factors influencing color stability and evaluating repair protocols tailored to specific material classes under clinically relevant conditions.

## 5. Conclusions

In this study, both manufacturing method and surface treatment protocol affected the repair performance of resin-matrix crown materials. Tribochemical silica coating with silanization yielded the highest micro-shear bond strength, indicating the effectiveness of combined mechanical and chemical surface conditioning. CAD/CAM materials showed significantly higher bond strength than additively manufactured materials. Within the exploratory optical analysis, aging increased color mismatch, and additively manufactured materials tended to show greater color changes than CAD/CAM materials. While tribochemical silica coating with silanization may improve repair bond strength, the optical stability of repaired restorations appears to be influenced mainly by the restorative material; however, further studies powered specifically for optical outcomes are needed to confirm these findings.

## Figures and Tables

**Table 1:** Materials used in the study, including manufacturer, material type, composition, manufacturing method, and lot number.

Material	Manufacturer	Type	Composition	Manufacturing Method	Batch Number
**HIPC Plus**	Bredent GmbH, Senden, Germany	CAD/CAM polymer composite	Highly cross-linked polymer matrix reinforced with ceramic fillers	Subtractive (CAD/CAM milling)	567845
**GC Cerasmart**	GC Corp., Tokyo, Japan	Nanohybrid resin-ceramic	Resin matrix with nano-ceramic fillers (barium glass and silica)	Subtractive (CAD/CAM milling)	2408287
**VarseoSmile TriniQ**	BEGO, Bremen, Germany	Permanent crown resin	Methacrylate-based photopolymer resin with inorganic fillers	Additive (3D printing)	602284
**CrownTec**	Saremco Dental, Rebstein, Switzerland	Permanent crown resin	Methacrylate-based photopolymer resin with ceramic fillers	Additive (3D printing)	F654

**Table 2:** Mean micro-shear bond strength (MPa) and standard deviation values according to material type and surface treatment protocol. Statistically significant differences are indicated by * *p* < 0.05.

Source	F	*p*	η^2^
**Material**	198.880	<0.001 *	0.847
**Surface treatment**	193.876	<0.001 *	0.782
**Material × Surface treatment**	12.739	<0.001 *	0.414

**Table 3:** Mean micro-shear bond strength (µSBS) values (MPa) expressed as mean ± standard deviation according to material type and surface treatment protocol (n = 10 per subgroup). Different superscript letters within each column indicate statistically significant differences among surface treatments based on Tukey’s post hoc test (*p* < 0.05). CT: CrownTec; GC: GC Cerasmart; HIPC: HIPC Plus; VST: VarseoSmile TriniQ; APA: airborne particle abrasion; LT: laser treatment; TSC: tribochemical silica coating.

Surface Treatment	CT (MPa)	GC (MPa)	HIPC (MPa)	VST (MPa)
**APA**	12.434 ± 0.529 ^c^	18.302 ± 1.457 ^a^	16.494 ± 1.546 ^b^	12.594 ± 0.508 ^c^
**LT**	13.977 ± 0.717 ^c^	16.101 ± 0.386 ^b^	17.822 ± 0.530 ^a^	13.306 ± 0.197 ^c^
**TSC**	17.527 ± 0.470 ^a^	19.840 ± 0.721 ^a^	20.087 ± 1.349 ^a^	16.067 ± 0.455 ^b^
**Total**	14.646 ± 2.240	18.081 ± 1.817	18.134 ± 1.916	13.989 ± 1.574

**Table 4:** Distribution of failure modes according to material type and surface treatment. Data are presented as frequency (n) and percentage (%). Failure modes were classified as adhesive (A), cohesive (C), and mixed (M). CT: CrownTec; GC: GC Cerasmart; HIPC: HIPC Plus; VST: VarseoSmile TriniQ; APA: airborne particle abrasion; LT: laser treatment; TSC: tribochemical silica coating.

Material	Adhesive n (%)	Cohesive n (%)	Mixed n (%)
**CT**	2 (6.7)	8 (26.7)	20 (66.7)
**GC**	2 (6.7)	10 (33.3)	18 (60.0)
**HIPC**	2 (6.7)	7 (23.3)	21 (70.0)
**VST**	0 (0.0)	5 (16.7)	25 (83.3)
**Surface treatment**			
**APA**	3 (7.5)	8 (20.0)	29 (72.5)
**LT**	2 (5.0)	6 (15.0)	32 (80.0)
**TSC**	1 (2.5)	16 (40.0)	23 (57.5)

**Table 5:** Two-way ANOVA results for color mismatch (ΔE00) at baseline (T0) and after aging (T1), showing the effects of material type, surface treatment, and their interaction. Statistically significant differences are indicated by * *p* < 0.05.

Source	F (T0)	*p* (T0)	η^2^ (T0)	F (T1)	*p* (T1)	η^2^ (T1)
**Material**	0.033	0.992	0.001	1.097	0.354	0.030
**Surface treatment**	0.217	0.806	0.004	0.630	0.534	0.012
**Material × Surface treatment**	3.209	0.006 *	0.151	0.805	0.568	0.043

## Data Availability

The original contributions presented in this study are included in the article. Further inquiries can be directed to the corresponding author.

## Abbreviations

The following abbreviations are used in this manuscript:

APA	Airborne Particle Abrasion
CAD/CAM	Computer-Aided Design/Computer-Aided Manufacturing
CT	CrownTec
ΔE00	Color difference (CIEDE2000 formula)
ΔE00_mismatch	Color mismatch between repair and substrate
ΔE00_RepairChange	Color change of repair area (T1 vs. T0)
ΔE00_SubChange	Color change of substrate area (T1 vs. T0)
GC	GC Cerasmart
HIPC	HIPC Plus
LT	Laser Treatment
µSBS	Micro-shear Bond Strength
T0	Baseline measurement
T1	Post-aging measurement
TSC	Tribochemical Silica Coating
VST	VarseoSmile TriniQ
